# A New Strategy for Mapping Epitopes of LACK and PEPCK Proteins of *Leishmania amazonensis* Specific for Major Histocompatibility Complex Class I

**DOI:** 10.3390/ijms24065972

**Published:** 2023-03-22

**Authors:** Edlainne Pinheiro Ferreira-Sena, Daiana de Jesus Hardoim, Flavia de Oliveira Cardoso, Luiz Ney d’Escoffier, Isabela Ferreira Soares, João Pedro Rangel da Silva Carvalho, Ricardo Almir Angnes, Stenio Perdigão Fragoso, Carlos Roberto Alves, Salvatore Giovanni De-Simone, Josué da Costa Lima-Junior, Alvaro Luiz Bertho, Tânia Zaverucha-do-Valle, Franklin Souza da Silva, Kátia da Silva Calabrese

**Affiliations:** 1 Laboratório de Imunomodulação e Protozoologia, Instituto Oswaldo Cruz, Fundação Oswaldo Cruz, Rio de Janeiro 21040-360, RJ, Brazil; 2 Laboratório de Imunoparasitologia, Instituto Oswaldo Cruz, Fundação Oswaldo Cruz, Rio de Janeiro 21040-360, RJ, Brazil; 3 Laboratório de Bioquímica de Proteínas e Peptídeos, Centro de Desenvolvimento Tecnológico em Saúde, Fundação Oswaldo Cruz, Rio de Janeiro 21040-360, RJ, Brazil; 4 Laboratório de Síntese Química, Instituto de Biologia Molecular do Paraná, Curitiba 81350-010, PR, Brazil; 5 Laboratório de Biologia Molecular e Sistêmica de Tripanossomatídeos, Instituto Carlos Chagas, Fundação Oswaldo Cruz, Curitiba 81350-010, PR, Brazil; 6 Laboratório de Biologia Molecular e Doenças Endêmicas, Instituto Oswaldo Cruz, Fundação Oswaldo Cruz, Rio de Janeiro 21040-360, RJ, Brazil; 7 Center for Technological Development in Health (CDTS)/National Institute of Science and Technology for Innovation in Diseases of Neglected Populations (INCT-IDPN), Oswaldo Cruz Foundation (FIOCRUZ), Rio de Janeiro 21040-900, RJ, Brazil; 8 Laboratory of Epidemiology and Molecular Systematics (LESM), Oswaldo Cruz Institute, Oswaldo Cruz Foundation (FIOCRUZ), Rio de Janeiro 21040-900, RJ, Brazil; 9 Post-Graduation Program in Science and Biotechnology, Department of Molecular and Cellular Biology, Biology Institute, Federal Fluminense University, Niterói 22040-036, RJ, Brazil; 10 Plataforma de Citometria, Instituto Oswaldo Cruz, Fundação Oswaldo Cruz, Rio de Janeiro 21040-360, RJ, Brazil; 11 Centro de Desenvolvimento Tecnológico em Saúde, Fundação Oswaldo Cruz, Rio de Janeiro 21040-360, RJ, Brazil; 12 Faculdade de Biologia e Ciências da Saúde, Universidade Iguaçu, Dom Rodrigo, Nova Iguaçu, Rio de Janeiro 26275-580, RJ, Brazil

**Keywords:** cutaneous leishmaniasis, T epitopes, peptides, vaccine, bioinformatics, HLA, H2Db, MHC I, tetramer

## Abstract

Leishmaniasis represents a complex of diseases with a broad clinical spectrum and epidemiological diversity, considered a major public health problem. Although there is treatment, there are still no vaccines for cutaneous leishmaniasis. Because *Leishmania* spp. is an intracellular protozoan with several escape mechanisms, a vaccine must provoke cellular and humoral immune responses. Previously, we identified the *Leishmania* homolog of receptors for activated C kinase (LACK) and phosphoenolpyruvate carboxykinase (PEPCK) proteins as strong immunogens and candidates for the development of a vaccine strategy. The present work focuses on the in silico prediction and characterization of antigenic epitopes that might interact with mice or human major histocompatibility complex class I. After immunogenicity prediction on the Immune Epitope Database (IEDB) and the Database of MHC Ligands and Peptide Motifs (SYFPEITHI), 26 peptides were selected for interaction assays with infected mouse lymphocytes by flow cytometry and ELISpot. This strategy identified nine antigenic peptides (pL1-H2, pPL3-H2, pL10-HLA, pP13-H2, pP14-H2, pP15-H2, pP16-H2, pP17-H2, pP18-H2, pP26-HLA), which are strong candidates for developing a peptide vaccine against leishmaniasis.

## 1. Introduction

Leishmaniases represents a complex of diseases with a broad clinical spectrum and epidemiological diversity, considered a major public health problem. Ninety-two countries or territories are considered endemic or have reported cases of cutaneous leishmaniasis, while visceral leishmaniasis is present in 83 countries. Currently, more than 1 billion people living in endemic areas are at risk of infection. It is estimated that 30,000 new cases of visceral leishmaniasis and more than 1 million new cases of cutaneous leishmaniasis occur annually [1]. Although several attempts have been made to obtain vaccines against leishmaniasis, no product is suitable for human use [2]. Furthermore, treatment issues due to high toxicity, low compliance, and cases of resistance, together with a lack of efficiency in control measures, argue that a vaccine would be the most effective, safe, comprehensive, and economically viable way to control this endemic [3].

Immunological findings against *Leishmania amazonensis* infection in the murine model highlight is a strong tool for proposing immunology profiles and vaccines for cutaneous leishmaniasis [4,5]. In this context, previous studies with subcellular fractionation of *L. amazonensis* promastigote forms have shown that the microsomal fraction (FMic) associated with immunomodulators provides protection against experimental infection by this parasite [6]. Furthermore, analyses of the different components of the fraction allowed for the identification of *Leishmania* spp. The homolog of receptors for activated C kinase (LACK) and phosphoenolpyruvate carboxykinase (PEPCK) proteins are responsible for the observed protection [7], suggesting that the production of recombinant forms of peptides of these proteins could lead to the development of a vaccine against cutaneous leishmaniasis.

In recent years, several *Leishmania* antigens have been analyzed as vaccine candidates with different protective immune responses in various experimental models [8,9,10]. One strategy that can elicit a strong immune response is the identification and use of peptides containing binding epitopes of the major histocompatibility complex (MHC) [11]. It is known that proteins have several immunodominant epitopes that can induce immunity through presentation via MHC proteins present on the surface of antigen-presenting cells (APCs) [12]. Thus, the identification of these epitopes can be efficient in the development of epitope-based vaccines. Several studies have also revealed that the strategy of epitope-based vaccines, chosen for their binding to human leukocyte antigen, appears to induce more potent responses than whole-antigen vaccines [13].

Using peptides containing immunogenic parts of a protein capable of inducing a specific T-cell response may become a promising strategy in the prophylaxis of leishmaniasis [13,14,15]. In addition, the development and use of bioinformatics tools have made it easier to identify potential T-cell epitopes restricted to human leukocyte antigens (HLA) and murine (H2) immunogenic vaccines. Peptide-based vaccines have advantages, including the absence of infectious materials, specificity, stability, and large-scale production at low cost, and these peptide vaccines have already been successfully tested against several diseases [16,17,18,19,20]. In addition, potential immunogenic peptides have already been identified within proteins and described as vaccine candidates. For example, *Leishmania* histone H2B, Promastigote Surface Antigen (PSA), and *Leishmania major* L. form-large RAB GTPase (LmlRAB) induced a predominant Th1 response in individuals immune to *L. major* or *L. infantum* [21,22,23].

Therefore, possible immunogenic peptides can be identified within proteins previously described as vaccine candidates. This work identifies potential antigenic peptides from LACK and PEPCK *L. amazonensis* proteins from a workflow that includes in silico and in vitro approaches. In addition, CD8+ T-lymphocyte epitopes (n = 3 and n = 6, respectively), capable of binding to MHC class I proteins, were identified and designed to build antigenic peptides as vaccine targets. The set of results presented here on antigenic epitope mapping of these proteins contributes to improving the rational development of vaccines based on specific regions of these proteins that can trigger protective immune responses.

## 2. Results

### 2.1. Promiscuity Analysis of Human and Murine MHC I Alleles

The distribution of the HLA allele varies among populations, but vaccinal peptides need to be immunogenic for most individuals. The Brazilian population is highly miscegenated, but migration history is different for each region. Therefore, before starting antigenic prediction, the Allele Frequency Net Database was searched for the most frequent allele in each Brazilian state. As a result, HLA-A*0201 was chosen for its high frequency compared to other types of HLA throughout the Brazilian territory. In parallel, H2Db was the allele chosen for predicting immunogenic epitopes because it can be found in C57BL/6J mice [24], a representative strain in various life science fields. In addition, it is frequently used for transgenesis, being permissive for the expression of most mutations, and it was the first strain for which the mouse genome was sequenced [25].

### 2.2. Prediction of LACK and PEPCK Epitopes

*Leishmania major* immunogenic peptides already deposited in the Immune Epitope Database (IEDB) were used as positive controls to test the prediction parameters. Initially, the amino acid sequences were aligned between the LACK of *L. amazonensis* and *L. major* to find the similarity between sequences already known in the database with our predicted sequences (Supplementary Figure S1). Then, the proteins were subjected to two prediction strategies: (i) IEDB server—binding of 11 amino acid peptides with MHC-I in six algorithms without proteasomal cleavage, as the server does not allow it and (ii) Database of MHC Ligands and Peptide Motifs (SYFPEITHI) server—binding of 10 amino acid peptides with MHC-I with proteasomal cleavage. The use of servers with and without proteasomal cleavage increased the analysis range and provided greater reliability in the prediction and evaluation of the servers’ applicability.

**Figure 1:**
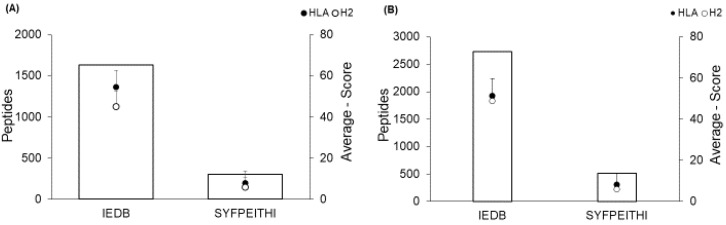
Number of predicted epitopes with major histocompatibility complex (MHC) class I interaction for murine H2Db and human HLAA*0201 haplotypes. Prediction of of the homolog of receptors for activated C kinase (LACK) (**A**) and phosphoenolpyruvate carboxykinase (PEPCK) (**B**) epitopes in the IEDB and SYFPEITHI servers. The histogram shows the number of peptides (left axis), and the bullets show each server’s mean scores ± SD (right axis).

The LACK epitope prediction generated the same number of possible candidates for binding to MHC-I in the H2Db and HLA-A*0201 haplotypes (IEDB, n = 1.632) and (SYFPEITHI, n = 303), although the mean scores were different for both H2Db (54.49 ± 7.62—IEDB; 7.86 ± 4.79—SYFPEITHI) and HLA-A*0201 (45.07 ± 8.05—IEDB; 5.75 ± 5.87). The same was observed in predicting PEPCK epitopes (IEDB, n= 2730) and (SYFPEITHI, n = 516), including the difference in mean H2Db score (48.79 ± 7.76—IEDB; 5.91 ± 4.82—SYFPEITHI) and HLA-A*0201 (51.25 ± 8.58—IEDB; 8.11 ± 5.58—SYFPEITHI) (Figure 1). From this prediction, 11.816 epitopes were totaled from the two proteins and the haplotypes.

**Figure 2:**
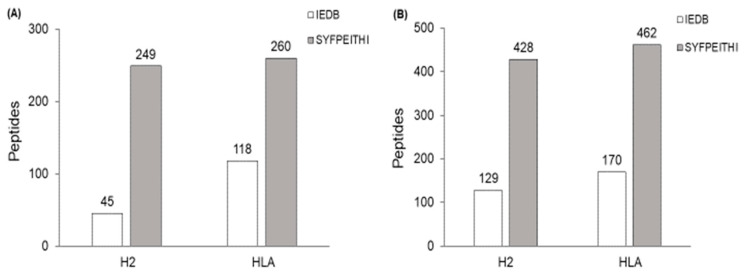
**Epitope selection by the score value.** Number of peptides predicted for LACK (**A**) and PEPCK (**B**) proteins after filtering by score value. Only peptides that reached scores ≥ 50% in at least 4 of the 6 IEDB servers and ≥2 for the SYFPEITH server were selected for the next phase.

To filter potential epitopes, we selected peptides with a score ≥ 50% in at least 4 different algorithms on the IEDB server and a score ≥ 2 on the SYFPEITHI server. From these parameters, the number of epitopes of LACK [H2Db (IEDB—97.0% and SYFPEITHI—17.8%) and HLA-A*0201 (IEDB—93.0% and SYFPEITHI—14%)] and PEPCK [H2Db (IEDB—95.0% and SYFPEITHI—17.0%) and HLA-A*0201 (IEDB—93.7% and SYFPEITHI—10.5%)] were reduced (Figure 2).

To identify peptides with a conserved motif in the two servers, the total predicted peptides of LACK (H2Db and HLAA*201) and PEPCK (H2Db and HLAA*201) were submitted separately to the MEME Suite server. First, the residues that appeared the most in the set of peptides were chosen. Then, peptides that presented conserved amino acid residues in the logo plot and E-value ≤ 2 were selectedThe selected peptides followed the criteria of conserved amino acid residues in the logo plot and presented the best E-values. LACK H2 epitopes were the only ones giving conserved motifs (LEHPIVV and GAKPSECI). Therefore, LACK HLA PEPCK epitopes for H2 (ENVEWG and ELVQWA) and HLA (GGCYAK and VLSYAK) were selected by E-value (Supplementary Figure S2 and Table S1). Then, the motifs were aligned in the multialign tool to identify anchor amino acid residues in the peptides (Supplementary Figure S3). Finally, 26 peptides were selected (Table 1).

### 2.3. Prediction of Physicochemical Parameters

The ProtParam server showed physicochemical results of the epitopes alone, where the molecular weight, theoretical protrusion index (P1), estimated in vitro half-life in human mammalian reticulocytes, and the instability index was calculated, revealing that all peptides can be stable for vaccine targets. In addition, the aliphatic calculated index indicated a thermostable nature at different temperatures and the general average of hydropathicity (GRAVY) (Table 2).

### 2.4. Validation of Predicted Epitopes in Spleen Cells from Infected Mice by ELISpot

The 26 selected sequences were produced as synthetic peptides to confirm the immunogenicity of the predicted epitopes. After synthesis, the peptides were purified in HPLC coupled to mass spectrometry (Supplementary Figure S4). Of the 26 peptides, the peptide pP25-HLA was lost during purification and was not tested.

**Figure 3:**
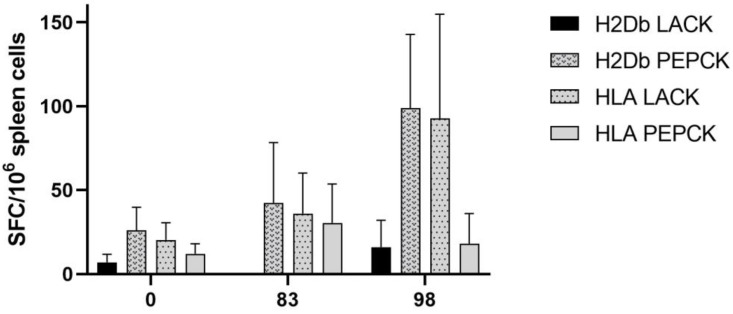
**Interferon-γ** (IFN-γ) **producing cells induced by peptide stimulation.** Peptides were pooled according to protein and MHC origin and interacted with splenocytes from *L. amazonensis* infected C57BL/6 mice (83 and 98 days after infection), and non-infected mice (0 days). Interferon-producing cells were estimated by ELISpot.

Specific T-cell responses, measured by the production of interferon-γ (IFN-γ) after interaction with spleen lymphocytes from *L. amazonensis*, were observed in infected and the control mice. The immunogenicity of the 25 synthetic peptides was initially validated by groups according to the protein of origin and MHC haplotype used on the prediction. Splenocytes derived from infected mice were collected 83 and 98 days after infection and were stimulated ex vivo using 4 pools of peptides: LACK murine (LACK–H2Db) and human (LACK–HLA-A*0201) haplotypes, and PEPCK murine (PEPCK–H2Db) and human (PEPCK–HLA-A*0201) haplotypes. PEPCK–H2Db and LACK–HLA-A*0201 peptides induced a specific IFN-γ response, especially 98 days after infection (Figure 3). No significant numbers of IFN-γ secreting cells were observed. All cells stimulated with ConA had high numbers of IFN-γ secreting cells, while control, non-stimulated cells presented no significant numbers of IFN-γ secreting cells.

### 2.5. Quantification of the T Lymphocyte Subpopulations Reactive to the Predicted Epitopes

**Figure 4:**
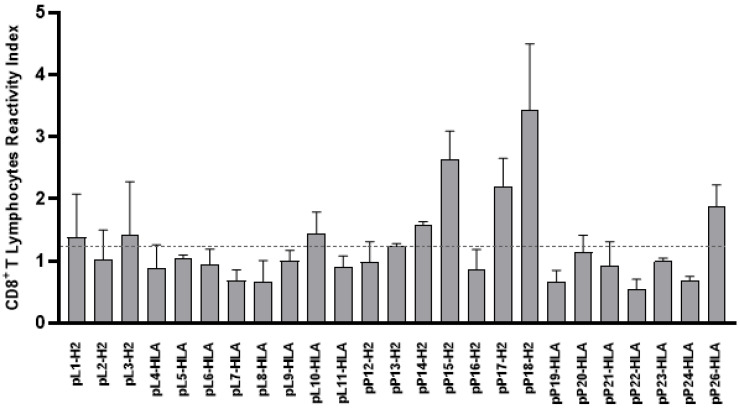
**CD8+ T lymphocyte reactivity with DimerX.** Assays were performed by flow cytometry and revealed the H2Db Ig/peptide complex. The experiments were conducted with a pool of cells from lymph nodes of *L. amazonensis* infected and non-infected mice. Each of the 25 peptides was coupled to DimerX-specific haplotype mouse cells. Cells were also marked with an anti-CD8 monoclonal antibody. The percentage of double-positive cells from infected mice was divided from the ratio found in cells from normal mice to determine the reactivity index shown in the figure. The horizontal line shows the 20% cutoff used to select peptides with greater specific interaction with infected lymphocytes.

The immunogenicity of the 25 synthetic peptides was individually validated by analyzing their ability to bind to lymph node cells from *L. amazonensis* infected mice. DimerX/peptide complexes were used in ex vivo assays to assess the percentage of CD3 T lymphocytes that would specifically bind to these supramolecular complexes, which mimic interactions that occur during antigen presentation. Cell/complex binding was measured by flow cytometry analysis. In tests with cells and DimerX-H2Db/peptide complexes, double staining was interpreted as clones of CD3 T lymphocytes, specifically reactive to the complexes and reactive CD8+ lymphocytes, capable of binding to DimerX-H2Db/peptide complexes (Supplementary Figure S5). Of the 25 reactive peptides to the DimerX/PEPTIDES complex, 8 gave a specific MHC class I binding response (Figure 4).

## 3. Discussion

Knowledge of the epitopes responsible for T-lymphocyte-mediated immunity is essential for vaccine studies in *Leishmania* spp. However, to date, most leishmaniasis vaccines have failed due to poor antigen response to selected proteins/peptides, lack of a strong and persistent cellular response, and lack of standardized guidelines for experimental assays [26,27]. To contribute to overcoming these limitations, this work advances into the predictive dissection of T-lymphocyte epitopes present in LACK and PEPCK virulence factors from *L. amazonensis*, pointing to improve the selection of antigenic determinants from both proteins as sources of potent epitopes.

One of the great challenges for developing vaccines against cutaneous leishmaniasis is the differences found among *Leishmania* species, even when they cause similar forms of the disease [9,28]. For instance, although lipophosphoglycan (LPG) is considered a virulence factor for *L. major*, the same does not seem to be the case for *L. amazonensis* [28]. Similarly, protection elicited with a Th1 response during *L. major* infection has already been established, whereas, in *L. amazonensis*, the disease can persist in the presence of Th1 response [29]. Therefore, different virulence factors and immune responses induced by other species must be considered when developing a vaccine. In parallel, the use of the whole parasite has been seen as safe and effective, but there is still a challenge in standardizing doses. Therefore, in conjunction with in vitro/ex vivo validation, an immunome-derived vaccine approach may accelerate the development of candidate vaccines for leishmaniasis [28,30,31,32,33].

Immunomics tools and databases have favored coping with this barrier as an accurate strategy in epitope mapping in *Leishmania* spp., as applied here [34]. Although some reports of epitope mapping have been the subject of current studies in reverse vaccinology in *Leishmania* spp. [35], there is still a need to establish a better strategy for promising mapping epitopes from *Leishmania* spp. proteins. Controversies about the activation pathway of CD8+ T-lymphocyte, concerning the presentation of *Leishmania* antigens through MHC class I [36,37], and the absence of consensus on the most appropriate strategy to predict true positive epitopes [38,39,40] must be overcome. In this context, the experimental design proposed here started forecasting with two quantitative matrix methods widely used in the literature: the Immune Epitope Database—IEDB, using different prediction methodologies within the platform itself, including Artificial Neural Network—ANN, Stabilized Matrix Method—SMM, SMMPMBEC, Pickpocket, CONSENSUS, and NetMHCpan; and the SYFPEITHI server. However, as these methods do not differentiate between ligands and non-ligands, the immunogenicity of these peptides must be carefully evaluated through quantitative methods. Score evaluation and haplotype selection are fundamental for choosing possible immunogenic epitopes.

Analysis of the protein sequences yielded many possible epitopes from both proteins, but only a few were selected from all the algorithms used, chosen based upon the efficiency binding to more than one supertype or allele. Peptides that had high and reliable scores were further selected. In addition, the motifs of these peptides and their proteasomal cleavage were observed. Based on these selections, and the MHC class I-restricted epitopes prediction that is considered reliable, we designed 26 synthetic peptides from the two *L. amazonensis* proteins. These peptides were then submitted to an experimental approach to validate their ability to bind and activate *Leishmania*-infected lymphocytes from MHC-compatible C57BL/6J mice, further confirmed by molecular docking. This approach narrowed our numbers to 9 selected peptides, capable of inducing IFN-γ production and binding to CD8+ lymphocytes through MHC class I. Developing a high throughput method for screening peptides for MHC binding to determine CD8+ T-lymphocyte responses, such as IFN-γ ELISpot and tetramer/peptide binding technologies, is a proof of concept for prediction validation.

LACK protein has long been identified as an important virulence factor of *Leishmania* spp. and a potential immunogen and drug target against the parasite [41]. Its sequence is highly conserved among *Leishmania* species [42], which encourages its exploitation for vaccine development. The data presented here corroborates the antigenic properties of LACK peptides previously proposed for other studies that evaluated the immune response of LACK epitopes as vaccine targets against *L. major*. Salehi-Sangani et al. showed that a chimeric protein that included the most immunogenic epitopes from the genes of several proteins, including LACK, induced a high level of IFN-γ, partially protecting mice against *L. major* [11]. Another study identified LACK peptides that are effectively presented via MHC class II molecules from mice of susceptible and resistant backgrounds [43]. Our results indicate that LACK peptides pL1H2, pL3H2, and pL10HLA are highly antigenic for *L. amazonensis*, all comprising sequences similar to those previously identified for *L. major* [11,43].

PEPCK is a key player in the gluconeogenesis pathway in *Leishmania* spp. [44]. Its homology among pathogenic species of *Leishmania* reaches more than 90%, whereas it has less than 17% of homology to mice and humans [45], making it an excellent source of vaccine immunogens. Previous results show that recombinant PEPCK can protect mice from *L. major* infection [45]. Similarly, a synthetic DNA vaccine encoding the PEPCK gene was able to elicit a protective response against *L. major*, generating specific T memory cells [46]. To our knowledge, no epitope screening protocol was previously applied to that protein, although a peptide tetramer (PEPCK_335-351_) was shown to bind to CD4^+^ T cells from *L. major*-infected mice. The present study identified pP13-H2, pP14-H2, pP15-H2, pP17-H2, pP18-H2, and pP26-HLA as strongly antigenic and capable of binding specific CD8+ T lymphocytes and inducing IFN-γ production.

There has been an understanding that the activation of CD8+ cells and IFN-γ production are important for protection against intracellular pathogens [47]. Studies show that CD8+ T-lymphocytes contribute to the destruction of *Leishmania*-infected cells by activating macrophages for oxidative explosion via cytokines produced upon antigen stimulation [48,49]. Our work shows that LACK and PEPCK peptides induce a CD8+ T-lymphocyte response with either human or murine MHC. Similarly, peptides obtained from the cysteine proteinase B from *L. amazonensis* could recognize the T cell receptor on the surface of CD8+ lymphocytes, indicating the presence of specific cells on the microenvironment of the draining lymph nodes of the lesion [50].

Studies have identified T cell epitopes using infected macrophages as APCs. They have revealed the existence of potential T lymphocyte epitopes restricted to HLA classes I and II in the amino-terminal region that could stimulate specific cellular immune responses in volunteers infected with *L. donovani* or *L. panamensis* [14]. Not only external or secreted *Leishmania* antigens can be presented in the context of MHC class I proteins, but intracellular proteins can be as well [26,51]. Agallou and collaborators showed that peptides from *L. infantum* induced a T-cell response mainly characterized by priming CD8+ T lymphocytes and IFN-γ production in immunized mice [52]. Our work shows that the T-cell receptor (TCR) recognized the processed peptides bound to MHC class I, and CD8+ T lymphocytes were identified by binding the TCR-MHC complex through its interaction with non-polymorphic regions of the MHC H2Db/peptide.

Despite the mechanism by which the MHC complex binds to peptides not being fully understood, the use of soluble MHC-peptide complexes and tetramers allows epitope mapping and detection of antigen-specific T-lymphocytes from *L. amazonensis* proteins [50,53]. The data gathered here present candidate peptides for vaccine development and advances new and powerful examples of tetramer use for *L. amazonensis* epitope mapping. Furthermore, a better understanding of the mechanism underlying the complex interaction of the MHC proteins, with peptides assessed, can drive us to future responses regarding the cellular and humoral immunity of *L. amazonensis* peptides.

## 4. Materials and Methods

### 4.1. Human Population Coverage and Murine MHC Class I Allele

The distribution of HLA alleles among the endemic population is essential for effective vaccine development. Therefore, the human population coverage analysis tool Allele Frequency Net Database (http://www.allelefrequencies.net (accessed on 1 July 2020)) [54] was used to identify the highest frequent Human Leukocyte Antigen (HLA) allele in the Brazilian population. The search was performed for each Brazilian state. Murine MHC class I binding allele (H2Db-Beta-2-microglobulin) was selected from the chosen strain, C57BL/6J—*Mus musculus*, in Mouse Haplotype Table [55].

### 4.2. Recovery of Peptide Sequences

LACK (LAMA_000011500) and PEPCK (LAMA_000546600) complete aminoacid sequences from *Leishmania amazonensis* [56] were selected and retrieved from TriTrypDB—Kinetoplastid Informatics Resources (https://tritrypdb.org (accessed on 1 November 2019)) in FASTA format. Both sequences were inserted in The National Center for Biotechnology Information—NCBI (ncbi.nlm.nih.gov (accessed on 1 August 2020)). Immune Epitope Database and Analysis Resource—IEDB (iedb.org (accessed on 1 August 2020)) was used to verify the peptide sequences. As there are already studies with the prediction of LACK protein epitopes identified and experimentally tested, we used the sequences of these peptides as a control for our starting point [43].

### 4.3. Prediction of T Lymphocyte Epitopes

The sequences of *L. amazonensis* LACK and PEPCK were submitted to the six servers of the Immune Epitope Database—IEDB platform (Artificial Neural Network—ANN, Stabilized Matrix Method—SMM, SMMPMBEC, Pickpocket, CONSENSUS, NetMHCpan)—(iedb.org (accessed on 1 November 2020)) [57] and the server SYFPEITHI—(syfpeithi.de (accessed on 1 February 2021)) [58] for prediction of T cell peptides (MHC class I). These servers have a database for MHC ligands and peptide motifs in humans and other animal species. In addition, the SYFPEITHI server also predicts proteasome-cleaved peptides, although their content is restricted to published data only. After prediction, peptides with scores ≥50 for at least 4 of the 6 IEDB servers, or ≥2 for the SYFPEITHI server, were selected. Next, those were submitted to the MEME Suite software (https://meme-suite.org/meme (accessed on 1 May 2021)) [59] for analysis of the most predominant amino acids, followed by alignment in the Multalin software (multalin.toulouse.inra.fr (accessed on 1 May 2021)) [60] to observe which peptides had added residues on the N and C terminal chains, and to select possible immunogenic epitopes.

### 4.4. Tap Transport/Proteasomal Cleavage

To predict antigen processing through the class I MHC presentation pathway, we used the NetCTL 1.2 server combined with the Tap transport/proteasomal cleavage tools (https://services.healthtech.dtu.dk/service.php?NetCTL-1.2 (accessed on 1 January 2022)). The method integrates the prediction of class I MHC peptide binding, C-terminal proteasomal cleavage, and TAP transport efficiency. The C-terminal cleavage weight was set to 0.15, the Tap transport efficiency was set to 0.05, and the epitope ID was set to 0.75 (default values) [61].

### 4.5. Physicochemical Parameters and Antigenicity Prediction

The physicochemical parameters (isoelectric point, in vitro and in vivo half-life, molecular weight, instability index, aliphatic index, and large mean hydropathicity—GRA VY) and peptide solubility were predicted using the ProtParam server. http://web.expasy.org/protparam/ (accessed on 1 March 2022)) [62]. The antigenicity index of the epitopes for H2Db and HLA0201 was determined using the VaxiJen server http://www.ddg-pharmfac.net/vaxijen/VaxiJen/VaxiJen.html (accessed on 1 March 2022)) [63] at a threshold of 0.5 (for parasite selected as target organism).

### 4.6. Synthesis of Peptides and Purification

The peptides were synthesized by the F-moc strategy in a synthesizer machine (Multipep1; Intavis Bioanalytical Instruments, Köelh, Germany) using the Tentagel^®^ resin as described elsewhere [64]. Peptides were purified by an HPLC coupled to mass spectrometry. The chromatographic column used was the XBridge BEH C18 from Waters, with a particle size of 2.7 μM and dimensions of 5 cm × 4.6 mm. The mobile phase was comprised of the following eluents: 0.05% formic acid in H_2_O (18 MΩ × cm) and 0.05% formic acid in acetonitrile. The equipment used was a Waters Autopurification System with a Waters 2545 binary gradient pump, a Waters 2998 diode array detector, and a Waters SQ Detector 2 electrospray ionization source mass detector (IBMP 002063). The diode array detector performed monitoring from 200–300 nm, and the mass detector served monitoring from *m*/*z* 450–1450 in positive mode. The injection volume used was 20 µL of a sample with an estimated 1 mg/mL concentration. The chromatography column was kept at room temperature.

### 4.7. Mice and Infections

C57BL/6J male mice, aged 4 to 5 weeks, were obtained from ICTB-FIOCRUZ and brought to experimental mouse facilities, where they were acclimatized for at least one week before being used in the experiments. Upon arrival, mice were randomly separated into cages with 4 animals each and maintained in microisolators at random columns and rows. Animals were fed with standard pellet food and water ad libitum and maintained with enrichment items, which were changed every week. All experiments were carried out with the approval of the Ethics Committee for the Use of Laboratory Animals of Instituto Oswaldo Cruz (CEUA-IOC) under number L-014/2019. Mice were infected with 1 × 10^5^ *L. amazonensis* amastigotes in the left, hind footpad. After infection, mice were observed weekly for signs of illness (lethargy, ruffled fur, weight loss, footpad swelling).

### 4.8. Enzyme-Linked Immunospot Assay (ELISpot)

The ELISpot assay was performed with spleen cells obtained from *L. amazonensis*-infected mice at 83 and 98 days after infection; five mice were each assayed in duplicates. Three non-infected mice were used each time, but the results are shown together as 0 days after infection. Cells (250,000 cells per well) were stimulated with one of 4 different pools (pool 1—H2Db LACK with 3 peptides, pool 2—H2Db PEPCK with 7 peptides, pool 3—HLA LACK with 8 peptides, pool 4—HLA PEPCK with 8 peptides) of peptides (20 μg/peptide) and a RPMI medium with 10% FBS (negative control) or concanavalin—A (Sigma Aldrich, St. Louis, MO, USA) (4 μg/mL) (positive control). The reaction was carried out using the ELISpot Plus: Mouse IFN-γ (ALP)—MABTECH kit, according to the manufacturer’s recommendations. Spots generated after stimulation were counted with Immunospot reader S6UV ultra (Cellular Technology Ltd., Cleveland, OH, USA). The number of adjusted IFN-γ, secreting cells per million spleen cells, was expressed as the mean number of spots induced by antigen subtracted by the number of spots caused in non-stimulated wells.

### 4.9. Flow Cytometric Detection of T Cells Binding Complexed with Recombinant Dimers of MHC Class I (H-2Db) Molecule

To select the peptides with higher affinity to bind T lymphocytes, each one was coupled to MHC class I and then put in contact with lymphocytes from *L. amazonensis*-infected mice. The dimeric MHC class I (H-2Db) was a fusion protein between mouse H-2Db and mouse IgG1, and the complex was performed using a DimerX I: Recombinant Soluble Dimeric Mouse H-2D[b]: Ig kit, according to the manufacturer’s protocol (BD Biosciences, San Jose, CA, USA). Next, 10 µg of each peptide was complexed with the MHC molecule by overnight incubation. On the next day, approximately 3.3 × 10^5^ mouse lymph node cells/tube were resuspended in 1% FCS-PBS buffer and incubated for 10 min at 4 °C with Mouse BD Fc Block™ (purified CD16/CD32). Then, DimerX/H-2D (1 µg/mL) loaded with one of the 25 desired peptides (0.9 µg/mL) was added to each tube and incubated for 50 min at 4 °C. After washing, PE-conjugated mAb A85-1 (anti-mouse IgG1) and PE-Cy5 conjugated anti-mouse CD3e were added to each tube and incubated for 30 min at 4 °C. Controls with no antibody and no control isotype, with each antibody separate, were also prepared. Ten thousand events were acquired using a CytoFLEX flow cytometer (Beckman Coulter, Brea, CA, USA). The following parameters were considered: forward scatter to evaluate the cellular size, side scatter to evaluate cellular complexity, and analysis of cell marker expression with fluorescence analysis. PE (BD Biosciences) and PE-Cy-5 (BD Biosciences) fluorescence were acquired through 585/42 and 690/50 BP, respectively, of the 488 nm-blue laser. Data analysis was performed using CytoExpert software v2.3 (Beckman Coulter, Brea, CA, USA), and Kaluza v.2.1 (Beckman Coulter, Brea, CA, USA) was used to generate the histograms and dot plots (Supplementary Materials).

## 5. Conclusions

In silico analysis and prediction techniques of synthetic peptide sequences that are highly conserved and promiscuously bound to murine or human MHC class I molecules make them candidate vaccines against leishmaniasis. These findings provide complementary data on mapping epitopes to *L. amazonensis* LACK and PEPCK proteins. Data gathered here demonstrate that combining immunoinformatics approaches with experimental validation yields the identification of nine antigenic peptides: pL1-H2, pL3-H2, pL10-HLA, pP13-H2, pP14-H2, pP15-H2, pP17-H2, pP18-H2, and pP26-HLA. Based on these results, future investigations will be carried out to verify the ability of the peptides to induce protection in murine models infected with *L. amazonensis*.

## Figures and Tables

**Table 1:** T-cell epitope prediction of LACK and PEPCK.

ID	Sequence	Score	Proteasomal Cleavage Score	Tap Transport Efficiency	Antigenicity
pL1-H2	SLEHPIVVSGS	58.8	0.4515	−23.190	0.7372
pL2-H2	PSLEHPIVVSG	53.9	0.9244	−0.1090	0.8165
pL3-H2	PDGAKPSECIS	78.7	0.0403	−0.9380	1.0405
pL4-HLA	YVSTVTVSPDG	61.5	0.6117	0.2170	1.1232
pL5-HLA	VSTVTVSPDGS	77.5	0.1775	−17.510	0.8993
pL6-HLA	TVTVSPDGSLC	92.5	0.0438	−21.660	1.5174
pL7-HLA	YIKVVSTSRDG	81	0.9537	15.890	1.2568
pL8-HLA	VSTSRDGTAIS	78.5	0.3099	−0.4710	1.4326
pL9-HLA	STSRDGTAISW	81.5	0.7774	0.5600	0.7833
pL10-HLA	IKVVSTSRDGT	74.5	0.1949	−16.670	1.1628
pL11-HLA	KVVSTSRDGT	2 *	0.1808	−10.740	1.4372
pP12-H2	VRENVEWGSVN	77.2	0.0844	−21.230	−0.1759
pP13-H2	TDDVRENVEWG	60.4	0.0418	−20.720	1.1467
pP14-H2	ENVEWGSVNVK	58.8	0.1463	−15.960	0.1724
pP15-H2	DDVRENVEWGS	62.2	0.9084	0.3370	1.2258
pP16-H2	PELVQWALKLE	75.2	0.7149	−0.1350	1.0838
pP17-H2	APELVQWALK	2 *	0.9779	0.6480	0.6729
pP18-H2	LTAPELVQWA	3 *	0.9543	0.8820	0.3878
pP19-HLA	VFNIEGGCYAK	61.5	0.7825	30.580	−0.0962
pP20-HLA	IEGGCYAKAIG	85.5	0.3468	−0.8130	0.9997
pP21-HLA	GGCYAKAIGLN	73	0.0299	−18.340	0.5172
pP22-HLA	RGALCVLSYAK	41	0.9094	29.170	−0.0153
pP23-HLA	LCVLSYAKTGR	85	0.1467	−0.6050	0.1837
pP24-HLA	CVLSYAKTGRS	66	0.2616	−12.820	0.1697
pP25-HLA	LCVLSYAKTG	7 *	0.1408	−0.6050	0.1645
pP26-HLA	ALCVLSYAKT	7 *	0.7315	0.6350	−0.2272

* Score SYFPEITHI.

**Table 2:** Physicochemical parameters of peptides.

ID	Aminoacids	Molecular Weight	pI	Half-Life M *	Half-Life Y **	Half-Life E ***	Instability	Aliphatic	GRAVY
pL1-H2	11	1124.26	5.22	1.9 h	>20 h	>10 h	−8.1	123.64	0.509
pL2-H2	11	1134.30	5.25	>20 h	>20 h	-	9.41	123.64	0.436
pL3-H2	11	1103.21	4.37	>20 h	>20 h	-	62.63	44.55	−0.664
pL4-HLA	11	1124.21	3.80	2.8 h	10 min	2 min	34.46	79.09	0.255
pL5-HLA	11	1048.12	3.80	100 h	>20 h	>10 h	34.46	79.09	0.300
pL6-HLA	11	1078.20	3.80	7.2 h	>20 h	>10 h	34.46	88.18	0.564
pL7-HLA	11	1224.38	8.59	2.8 h	10 min	2 min	11.16	88.18	−0.273
pL8-HLA	11	1093.16	5.81	100 h	>20 h	>10 h	18.88	70.91	−0.155
pL9-HLA	11	1180.24	5.55	1.9 h	>20 h	>10 h	18.88	44.55	−0.618
pL10-HLA	11	1162.31	8.75	20 h	30 min	>10 h	3.45	88.18	−0.218
pL11-HLA	10	1049.15	8.75	1.3 h	3 min	3 min	11.28	58.00	−0.690
pP12-H2	11	1288.38	4.53	100 h	>20 h	>10 h	−14.00	79.09	−0.727
pP13-H2	11	1319.35	3.92	7.2 h	>20 h	>10 h	−14.00	52.73	−1.418
pP14-H2	11	1260.37	4.53	1 h	30 min	>10 h	−16.62	79.09	−0.673
pP15-H2	11	1305.32	3.92	1.1 h	3 min	>10 h	−14.00	52.73	−1.427
pP16-H2	11	1325.57	4.53	>20 h	>20 h	-	−4.21	141.82	0.045
pP17-H2	10	1154.37	6.05	4.4 h	>20 h	>10 h	22.12	127.00	0.200
pP18-H2	10	1127.31	4.00	5.5 h	3 min	2 min	30.61	127.00	0.520
pP19-HLA	11	1200.37	5.96	100 h	>20 h	>10 h	121.73	70.91	0.255
pP20-HLA	11	1081.25	5.99	20 h	30 min	>10 h	81.78	89.09	0.473
pP21-HLA	11	1066.24	8.20	30 h	>20 h	>10 h	41.84	89.09	0.409
pP22-HLA	11	1180.43	9.31	1 h	2 min	2 min	8.33	115.45	0.636
pP23-HLA	11	1210.46	9.31	5.5 h	3 min	2 min	16.05	106.36	0.409
pP24-HLA	10	1184.38	9.31	1.2 h	>20 h	>10 h	55.99	70.91	−0.009
pP25-HLA	10	1054.27	8.20	5.5 h	3 min	2 min	16.65	117.00	0.900
pP26-HLA	10	1068.30	8.24	4.4 h	>20 h	>10 h	25.14	127.00	1.120

**Estimated half-life:** * (mammalian reticulocytes, in vitro), ** (yeast, in vivo), *** (*Escherichia coli*, in vivo).

## Data Availability

All data can be found within the article and its supplementary files.

## Supplementary Materials

The following supporting information can be downloaded at: https://www.mdpi.com/article/10.3390/ijms24065972/s1.
