# Supplementary material for: A New Strategy for Mapping Epitopes of LACK and PEPCK Proteins of Leishmania amazonensis Specific for Major Histocompatibility Complex Class I
Source: Int J Mol Sci. 2023 Mar 22;24(6):5972. doi: 10.3390/ijms24065972 (PMC10054446; doi:10.3390/ijms24065972)
Supplement: Supplementary file 1 [file ijms-24-05972-s001.zip › ijms-2156438-supplementary.pdf]

# Supplementary Material

## Supplementary Figure S1

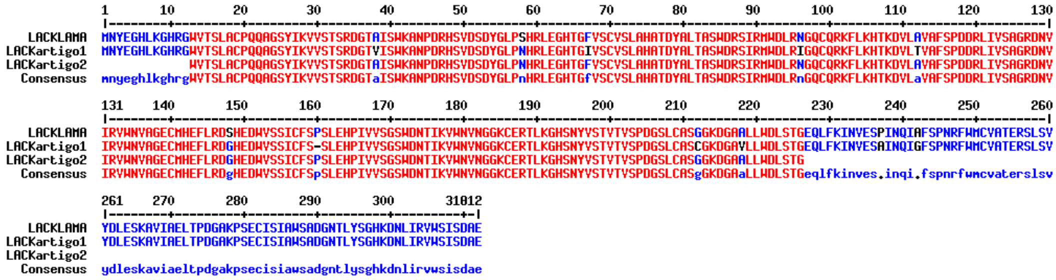


Homology between *L. amazonensis* and *L. major* LACK protein amino acids sequence present in TriTrypDB database.

**Supplementary Table S1**

**Epitopes selection on the MEME Suite server.**

**LACK**

| H2Db | Score | HLA0201 | Score |
|---|---|---|---|
| SLEHPIVVSGS | 58,8 | YVSTVTVSPDG | 61,5 |
| PSLEHPIVVSG | 53,9 | VSTVTVSPDGS | 77,5 |
| PDGAKPSECIS | 78,7 | TVTVSPDGSLC | 92,5 |
| PDGAKPSECI | 12,0 | YIKVVSTSRDG | 81,0 |
| PSLEHPIVVS | 6,0 | VSTSRDGTAIS | 78,5 |
| SLEHPIVVSG | 3,0 | STSRDGTAISW | 81,5 |
| DGAKPSECIS | 2,0 | IKVVSTSRDGT | 74,5 |
| | | VTVSPDGSLC | 5,0 |
| | | STVTVSPDGS | 7,0 |
| | | TVSPDGSLCA | 11,0 |
| | | TVTVSPDGSL | 15,0 |
| | | IKVVSTSRDG | 2,0 |
| | | STSRDGTAIS | 8,0 |
| | | VSTSRDGTAI | 9,0 |
| | | KVVSTSRDGT | 10,0 |
| | | VVSTSRDGTA | 11,0 |

**PEPCK**

| H2Db | Score | HLA0201 | Score |
|---|---|---|---|
| VRENVEWGSVN | 77,2 | VFNIEGGCYAK | 61,5 |
| TDDVRENVEWG | 60,4 | IEGGCYAKAIG | 85,5 |
| ENVEWGSVNVK | 58,8 | GGCYAKAIGLN | 73,0 |
| DDVRENVEWGS | 62,2 | RGALCVLSYAK | 41,0 |
| PELVQWALKLE | 75,2 | LCVLSYAKTGR | 85,0 |
| RENVEWGSVN | 2,0 | CVLSYAKTGRS | 66,0 |
| DDVRENVEWG | 5,0 | FNIEGGCYAK | 11,0 |
| VRENVEWGSV | 7,0 | GGCYAKAIGL | 12,0 |
| ENVEWGSVNV | 10,0 | IEGGCYAKAI | 17,0 |
| DVRENVEWGS | 10,0 | NIEGGCYAKA | 3,0 |
| APELVQWALK | 2,0 | LCVLSYAKTG | 7,0 |
| LTAPELVQWA | 3,0 | CVLSYAKTGR | 9,0 |
| ELVQWALKLE | 4,0 | GALCVLSYAK | 10,0 |
| PELVQWALKL | 13,0 | VLSYAKTGRS | 20,0 |
| TAPELVQWAL | 19,0 | ALCVLSYAKT | 7,0 |

**Supplementary Figure S2**

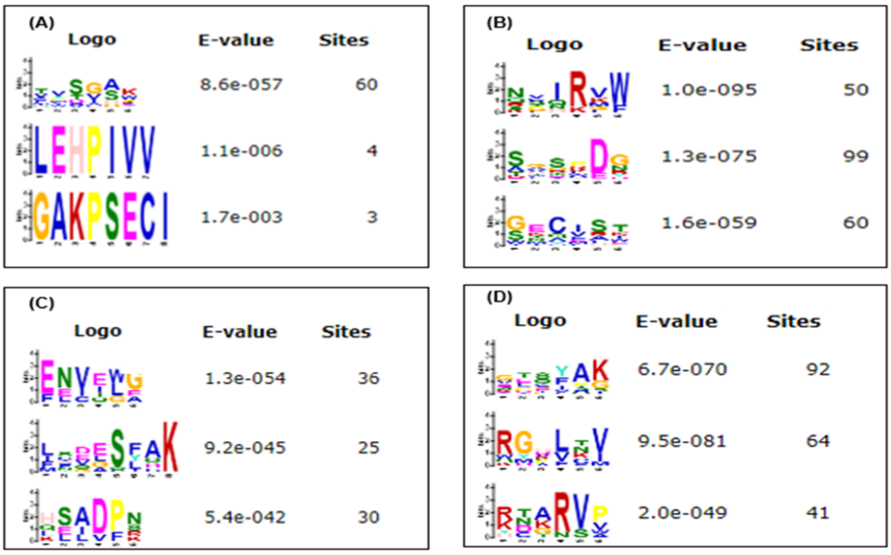


**Motif identification for the predicted LACK and PEPCK epitopes of the H2 and HLA haplotypes.** The LACK and PEPCK epitopes from the IEDB and SYFPEITHI servers were submitted to the MEME suite server. (A) Predicted LACK epitopes in H2Db. (B) Predicted epitopes of LACK in HLAA*0201. (C) Predicted epitopes of PEPCK in H2Db. (D) Predicted epitopes of PEPCK in HLAA*0201.

**Supplementary Figure S3**

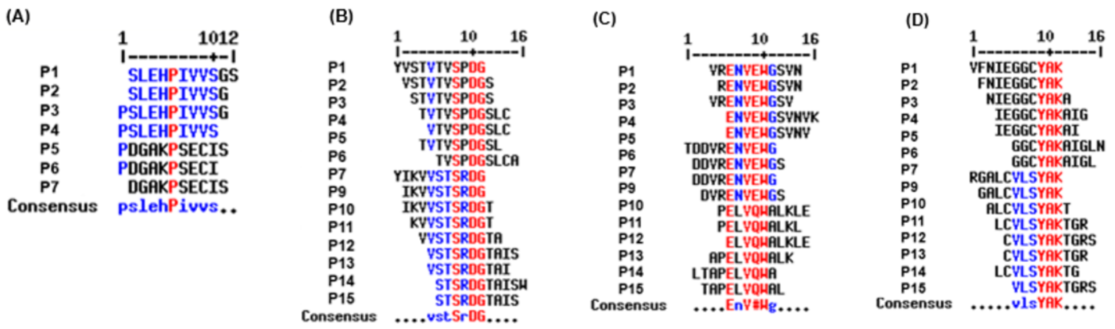

**Alignment of selected epitopes in MEME Suite.** (A) Predicted LACK epitopes in H2Db. (B) Predicted epitopes of LACK in HLAA*0201. (C) Predicted epitopes of PEPCK in H2Db. (D) Predicted epitopes of PEPCK in HLAA*0201.

## Supplementary Figure S4

## Chromatograms of peptide purifications

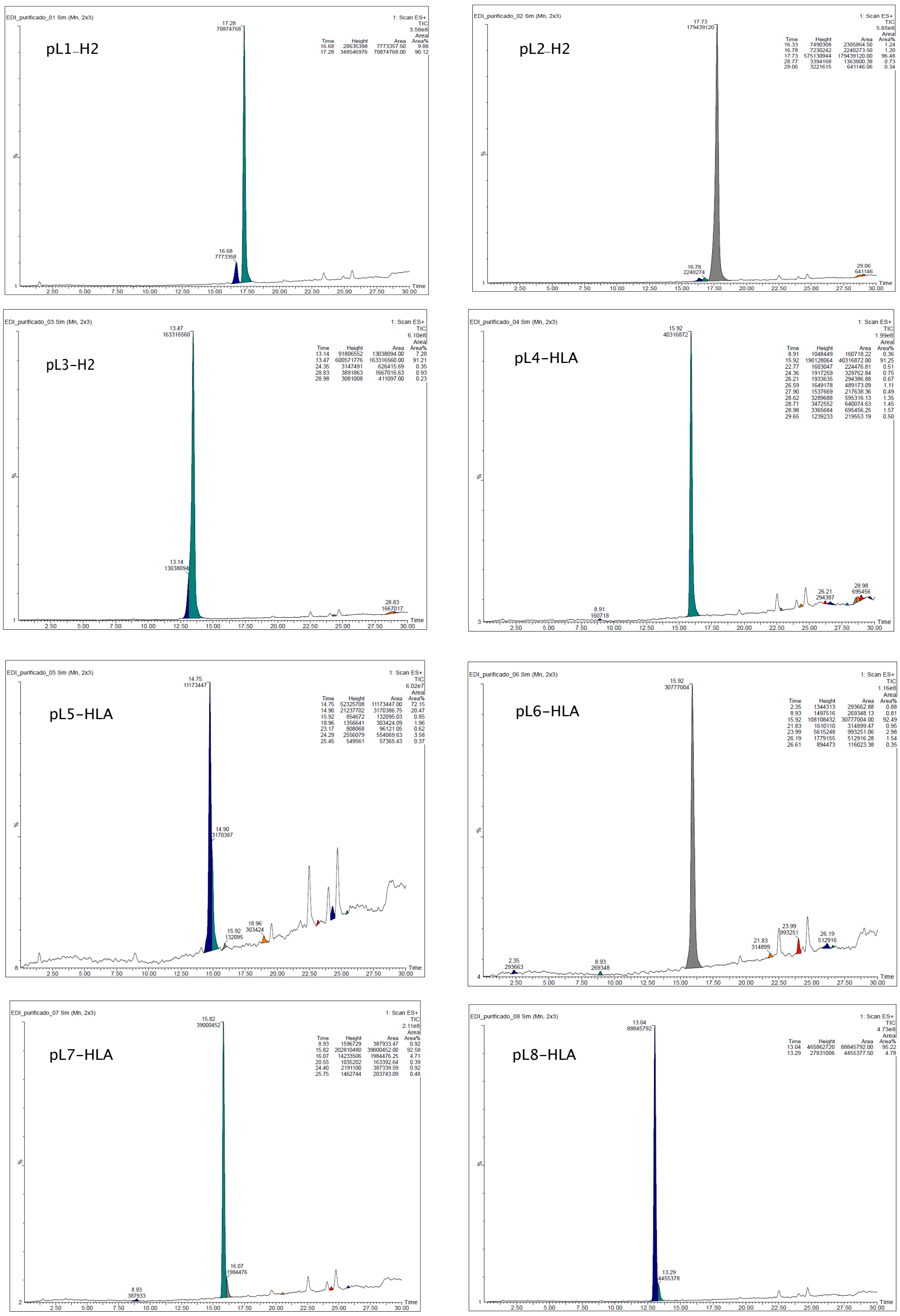

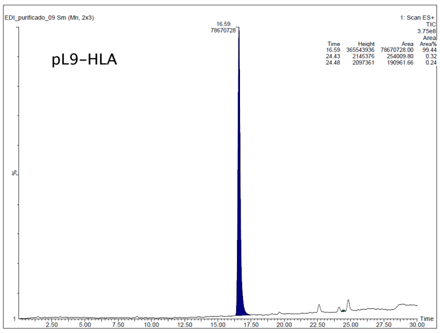
EDI_purificado_09 Sm (Mn, 2x3)
1: Scan ES+
TIC
3.75e8
pL9–HLA
16.59
78670728
Time Height Area Area%
16.59 365543936 78670728.00 99.44
24.43 2145376 254009.80 0.32
24.48 2097361 190961.66 0.24
%
Time

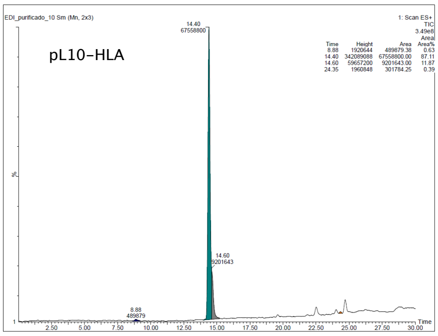
EDI_purificado_10 Sm (Mn, 2x3)
1: Scan ES+
TIC
3.49e8
pL10–HLA
14.40
67558800
Time Height Area Area%
8.88 1920644 489879.38 0.63
14.40 342089088 67558800.00 87.11
14.60 59657200 9201643.00 11.87
24.35 1960848 301784.25 0.39
14.60
9201643
8.88
489879
%
Time

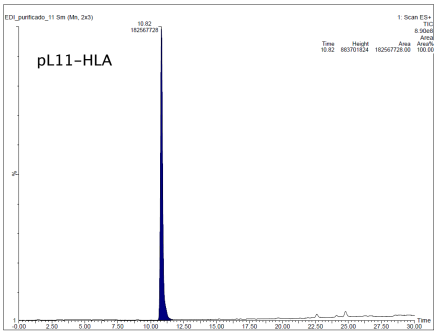
EDI_purificado_11 Sm (Mn, 2x3)
1: Scan ES+
TIC
8.90e8
pL11–HLA
10.82
182567728
Time Height Area Area%
10.82 883701824 182567728.00 100.00
%
Time

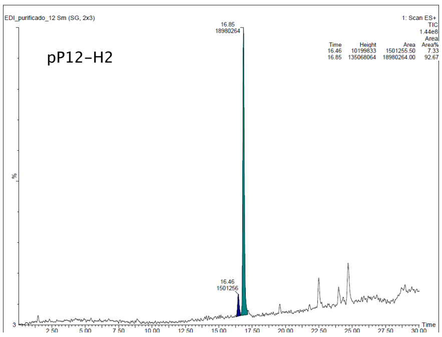
EDI_purificado_12 Sm (SG, 2x3)
1: Scan ES+
TIC
1.44e8
pP12–H2
16.85
18980264
Time Height Area Area%
16.46 10199833 1501255.50 7.33
16.85 135068064 18980264.00 92.67
16.46
1501256
%
Time

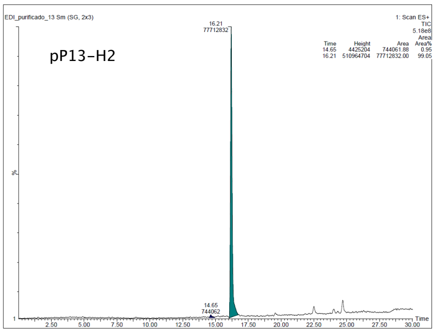
EDI_purificado_13 Sm (SG, 2x3)
1: Scan ES+
TIC
5.18e8
pP13–H2
16.21
77712832
Time Height Area Area%
14.65 4425204 744061.88 0.95
16.21 510964704 77712832.00 99.05
14.65
744062
%
Time

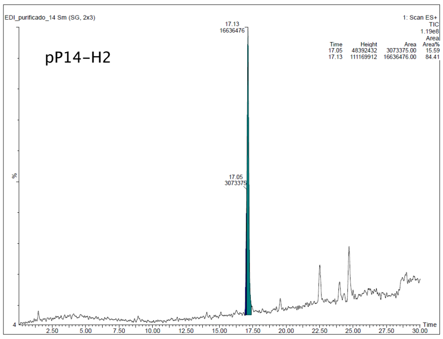
EDI_purificado_14 Sm (SG, 2x3)
1: Scan ES+
TIC
1.19e8
pP14–H2
17.13
16636476
Time Height Area Area%
17.05 48392432 3073375.00 15.59
17.13 111169912 16636476.00 84.41
17.05
3073375
%
Time

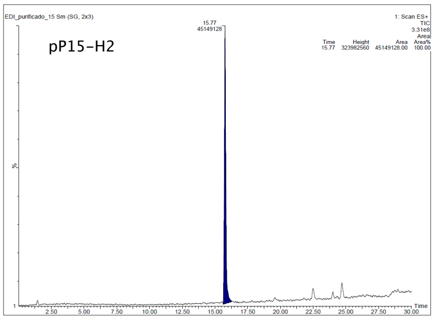
EDI_purificado_15 Sm (SG, 2x3)
1: Scan ES+
TIC
3.31e8
pP15–H2
15.77
45149128
Time Height Area Area%
15.77 323982560 45149128.00 100.00
%
Time

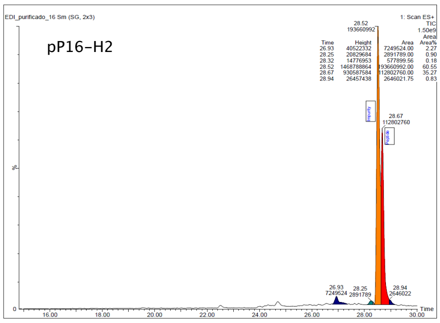
EDI_purificado_16 Sm (SG, 2x3)
1: Scan ES+
TIC
1.50e9
pP16–H2
28.52
193660992
Time Height Area Area%
26.93 40522332 7249524.00 2.27
28.25 20829684 2891789.00 0.90
28.32 14776953 577899.56 0.18
28.52 1468788864 193660992.00 60.55
28.67 930587584 112802760.00 35.27
28.94 26457438 2646021.75 0.83
Impurity
28.67
112802760
Peptide
26.93
7249524
28.25
2891789
28.94
2646022
%
Time

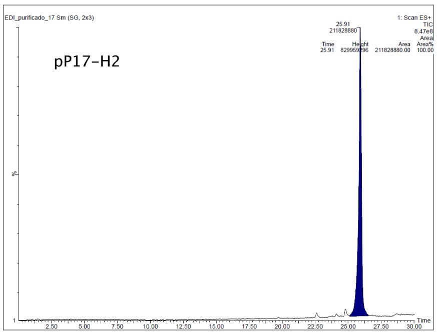
EDI_purificado_17 Sm (SG, 2x3)
1: Scan ES+ TIC 8.47e8
pP17-H2
25.91
211828880

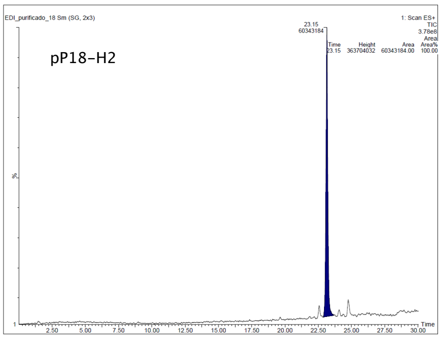
EDI_purificado_18 Sm (SG, 2x3)
1: Scan ES+ TIC 3.78e8
pP18-H2
23.15
60343184

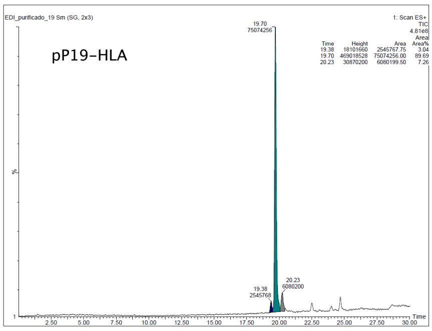
EDI_purificado_19 Sm (SG, 2x3)
1: Scan ES+ TIC 4.81e8
pP19-HLA
19.70
75074256

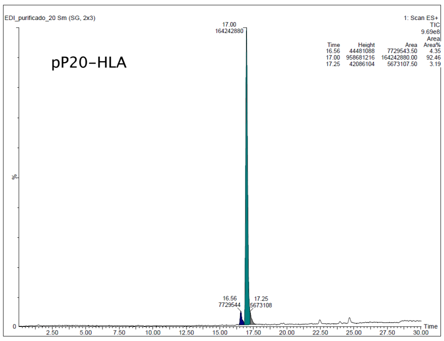
EDI_purificado_20 Sm (SG, 2x3)
1: Scan ES+ TIC 9.69e8
pP20-HLA
17.00
164242880

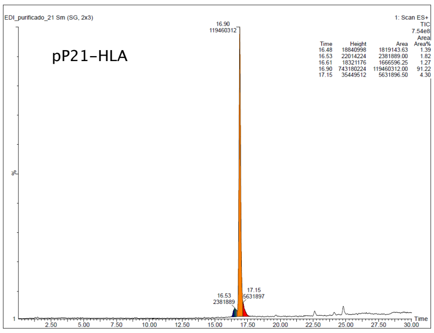
EDI_purificado_21 Sm (SG, 2x3)
1: Scan ES+ TIC 7.54e8
pP21-HLA
16.90
119460312

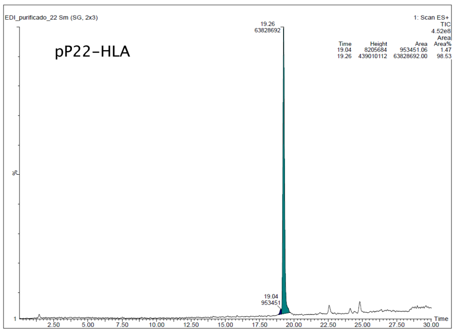
EDI_purificado_22 Sm (SG, 2x3)
1: Scan ES+ TIC 4.52e8
pP22-HLA
19.26
63828692

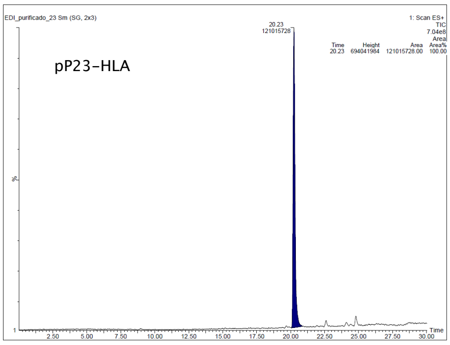
EDI_purificado_23 Sm (SG, 2x3)
1: Scan ES+ TIC 7.04e8
pP23-HLA
20.23
121015728

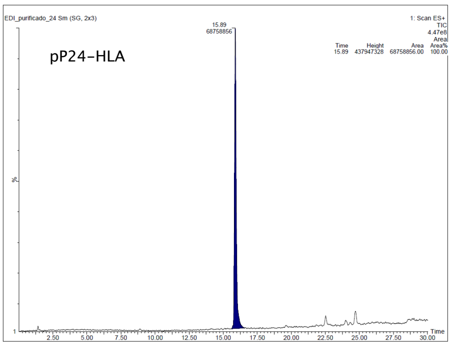
EDI_purificado_24 Sm (SG, 2x3)
1: Scan ES+ TIC 4.47e8
pP24-HLA
15.89
68758856

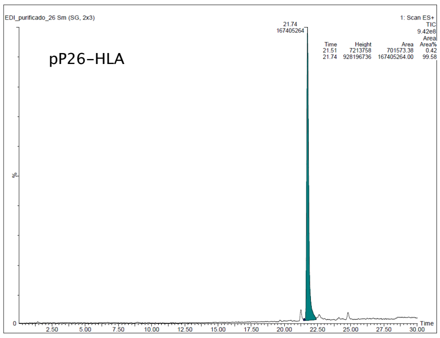
EDI_purificado_26 Sm (SG, 2x3)
1: Scan ES+
TIC
9.42e8
pP26-HLA
21.74
167405264
Time Height Area Area%
21.51 7213758 701573.38 0.42
21.74 928196736 167405264.00 99.58
%
0
2.50
5.00
7.50
10.00
12.50
15.00
17.50
20.00
22.50
25.00
27.50
30.00
Time

**Supplementary Figure S5**

**Cytometry gate analysis**

## Gate selection

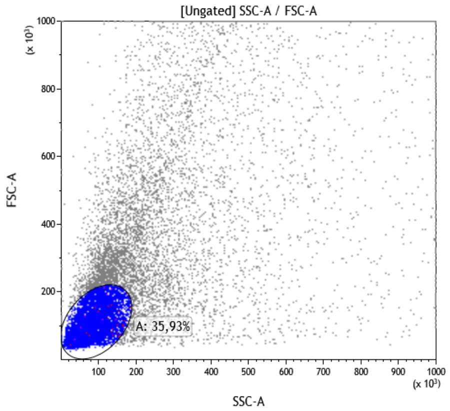


| Gate | %Total | %Gated | %GP Gated |
|---|---|---|---|
| All | 100,00 | 100,00 | N/A |
| A | 35,93 | 35,93 | N/A |

## Unmarked control

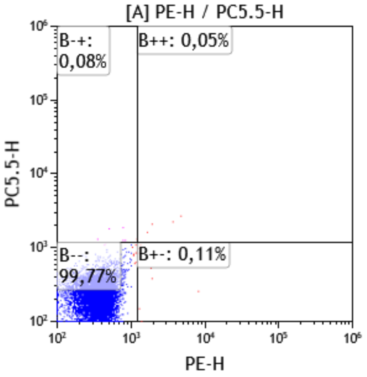


| Gate | Number | %Total | %Gated |
|---|---|---|---|
| All | 8.554 | 35,93 | 100,00 |
| B-- | 8.534 | 35,85 | 99,77 |
| B-+ | 7 | 0,03 | 0,08 |
| B+- | 9 | 0,04 | 0,11 |
| B++ | 4 | 0,02 | 0,05 |

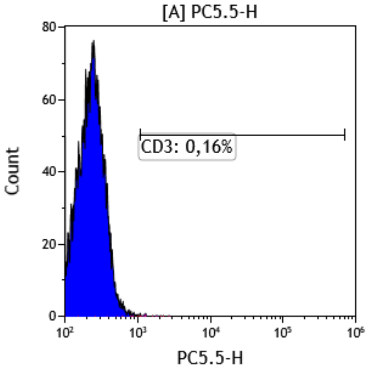


| Gate | Number | %Total | %Gated |
|---|---|---|---|
| All | 8.554 | 35,93 | 100,00 |
| CD3 | 14 | 0,06 | 0,16 |

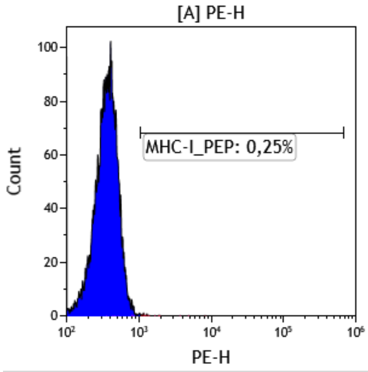


| Gate | Number | %Total | %Gated |
|---|---|---|---|
| All | 8.554 | 35,93 | 100,00 |
| MHC-I_PEP | 21 | 0,09 | 0,25 |

## Isotype Control

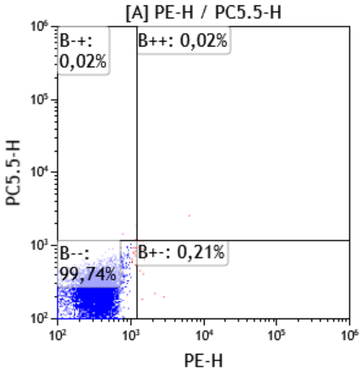


| Gate | Number | %Total | %Gated |
|---|---|---|---|
| All | 8.147 | 29,15 | 100,00 |
| B-- | 8.126 | 29,07 | 99,74 |
| B-+ | 2 | 0,01 | 0,02 |

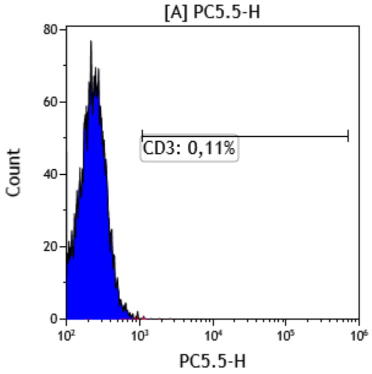


| Gate | Number | %Total | %Gated |
|---|---|---|---|
| All | 8.147 | 29,15 | 100,00 |
| CD3 | 9 | 0,03 | 0,11 |

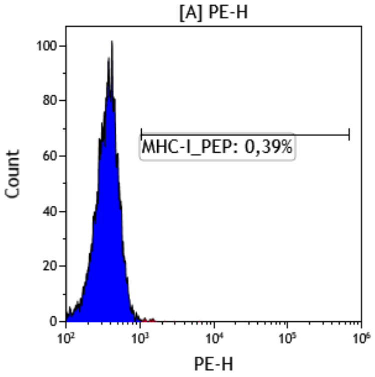


| Gate | Number | %Total | %Gated |
|---|---|---|---|
| All | 8.147 | 29,15 | 100,00 |
| MHC-I_PEP | 32 | 0,11 | 0,39 |

## CD3

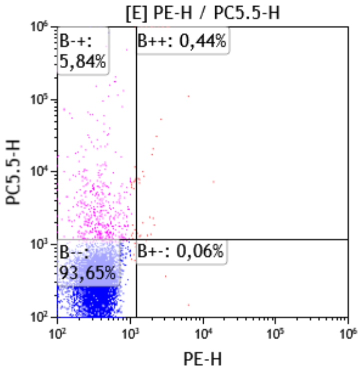


| Gate | Number | %Total | %Gated |
|---|---|---|---|
| All | 8.318 | 33,13 | 100,00 |
| B-- | 7.790 | 31,03 | 93,65 |
| B-+ | 486 | 1,94 | 5,84 |
| B+- | 5 | 0,02 | 0,06 |
| B++ | 37 | 0,15 | 0,44 |

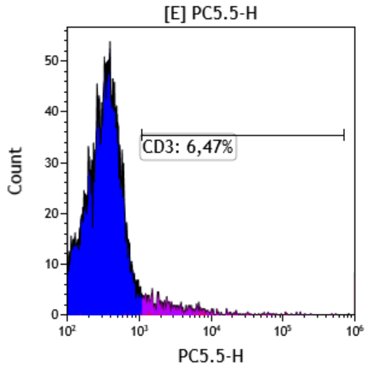


| Gate | Number | %Total | %Gated |
|---|---|---|---|
| All | 8.318 | 33,13 | 100,00 |
| CD3 | 538 | 2,14 | 6,47 |

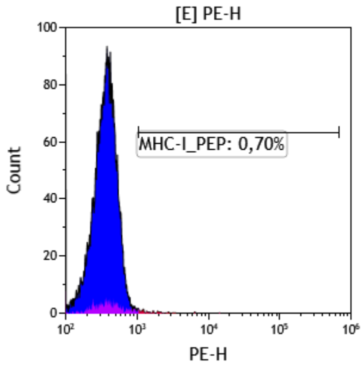


| Gate | Number | %Total | %Gated |
|---|---|---|---|
| All | 8.318 | 33,13 | 100,00 |
| MHC-I_PEP | 58 | 0,23 | 0,70 |

## MHC-I_PEP

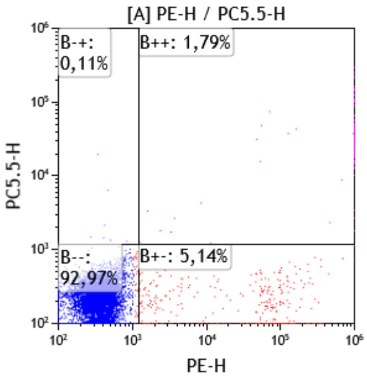


| Gate | Number | %Total | %Gated |
|---|---|---|---|
| All | 8.275 | 30,03 | 100,00 |
| B-- | 7.693 | 27,92 | 92,97 |
| B-+ | 9 | 0,03 | 0,11 |
| B+- | 425 | 1,54 | 5,14 |
| B++ | 148 | 0,54 | 1,79 |

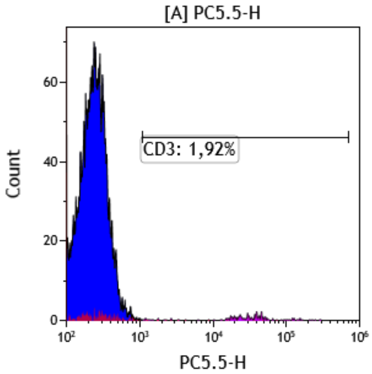


| Gate | Number | %Total | %Gated |
|---|---|---|---|
| All | 8.275 | 30,03 | 100,00 |
| CD3 | 159 | 0,58 | 1,92 |

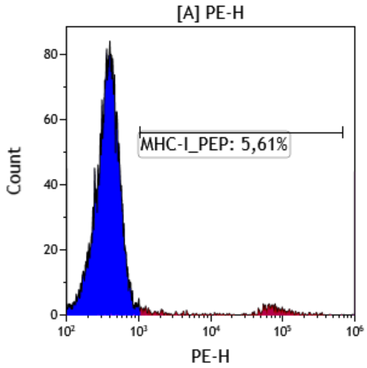


| Gate | Number | %Total | %Gated |
|---|---|---|---|
| All | 8.275 | 30,03 | 100,00 |
| MHC-I_PEP | 464 | 1,68 | 5,61 |
